# Prion Protein at the Leading Edge: Its Role in Cell Motility

**DOI:** 10.3390/ijms21186677

**Published:** 2020-09-12

**Authors:** Mariana Brandão Prado, Maria Isabel Melo Escobar, Rodrigo Nunes Alves, Bárbara Paranhos Coelho, Camila Felix de Lima Fernandes, Jacqueline Marcia Boccacino, Rebeca Piatniczka Iglesia, Marilene Hohmuth Lopes

**Affiliations:** Department of Cell and Developmental Biology, Institute of Biomedical Sciences, University of São Paulo, São Paulo 05508-000, Brazil; mariana.brandao.prado@usp.br (M.B.P.); isabelmelo@usp.br (M.I.M.E.); rodrigo.nunes.alves@usp.br (R.N.A.); coelhobarbarap@usp.br (B.P.C.); camila.felix@icb.usp.br (C.F.d.L.F.); jacquelineboccacino@usp.br (J.M.B.); rebecapi@usp.br (R.P.I.)

**Keywords:** prion protein, cell motility, adhesion, metastasis, invasiveness

## Abstract

Cell motility is a central process involved in fundamental biological phenomena during embryonic development, wound healing, immune surveillance, and cancer spreading. Cell movement is complex and dynamic and requires the coordinated activity of cytoskeletal, membrane, adhesion and extracellular proteins. Cellular prion protein (PrP^C^) has been implicated in distinct aspects of cell motility, including axonal growth, transendothelial migration, epithelial–mesenchymal transition, formation of *lamellipodia*, and tumor migration and invasion. The preferential location of PrP^C^ on cell membrane favors its function as a pivotal molecule in cell motile phenotype, being able to serve as a scaffold protein for extracellular matrix proteins, cell surface receptors, and cytoskeletal multiprotein complexes to modulate their activities in cellular movement. Evidence points to PrP^C^ mediating interactions of multiple key elements of cell motility at the intra- and extracellular levels, such as integrins and matrix proteins, also regulating cell adhesion molecule stability and cell adhesion cytoskeleton dynamics. Understanding the molecular mechanisms that govern cell motility is critical for tissue homeostasis, since uncontrolled cell movement results in pathological conditions such as developmental diseases and tumor dissemination. In this review, we discuss the relevant contribution of PrP^C^ in several aspects of cell motility, unveiling new insights into both PrP^C^ function and mechanism in a multifaceted manner either in physiological or pathological contexts.

## 1. Introduction

Cell motility is a fundamental process that entails intracellular remodeling, an intricate molecular network, and several cell–milieu interactions [1]. Motile cells are essential for several biological events, such as morphogenesis [2], angiogenesis [3], muscle contraction [4], vesicle transport [5], wound healing [6] and immune response, as well as for pathological events. Most cell types in different tissues are capable of migrating on a substrate, and cellular locomotion is governed by different processes [7]. Actin represents one of the main proteins in the cytoskeleton and its polymerization is key in the assembly and dynamic behavior of microfilaments [8]. Those microfilaments play a major role in the generation of cell polarity, since they are responsible for the formation of dynamic membrane protrusions of the cell edge, such as *lamellipodia*, *filopodia*, cell cortex, microvilli, as well as stress fibers that generate focal adhesions (FA) to the substratum, and in cytokinesis for cell division [9]. Cell migration capacity is of great importance not only for the correct function of the organism, but also for development, remodeling of adult tissue, and wound healing [10].

Abnormal cell motility contributes to a number of illnesses such as neuronal development disorders, immunodeficiency, cancer, and neurodegenerative diseases [11]. In cancer, the migration of tumor cells leads to invasion and metastasis—both processes being crucial for tumor development and poor response to treatment, and decisive for patient survival [12].

Cellular movement phenomena require the orchestration of numerous extra- and intracellular multiprotein complexes, and the cellular prion protein (PrP^C^), a cell membrane molecule, seems to have a pivotal role in cell movement in several aspects. PrP^C^ is encoded by the *PRNP* gene, and its expression is detected very early in the post-implantation embryo [13], being involved in the regulation of different mammalian stem and progenitor cell proliferation and differentiation mechanisms [14]. PrP^C^ is expressed in neurons and in most neural cell types, modulating astrocyte development [15], oligodendrocyte proliferation [16], and proper functioning of microglia in early stages [17]. Besides the central nervous system (CNS), PrP^C^ is also expressed in cells of the peripheral nervous system (PNS) [18], as well as in organs and tissues of other systems, such as the heart, intestine and pancreas [19].

PrP^C^ is organized into two main domains: a less flexible C-terminal domain of globular structure, with a glycophosphatidylinositol (GPI) membrane anchor [20,21,22], and a flexible and less organized N-terminal domain containing octapeptide repeats [23,24], closely related with the binding of PrP^C^ to a number of protein partners [25,26]. PrP^C^ is located on lipid rafts, which are cholesterol-rich portions of the cell surface highly associated with activation of signaling cascades [27], and can couple with plenty of membrane receptors located in these niches, forming multiprotein signaling platforms [27,28,29]. PrP^C^ ligands in the physiological and pathological contexts include transmembrane proteins, ion channels, extracellular matrix proteins and several secreted molecules including stress inducible protein 1 or heat shock organizing protein (STI1/HOP) [30,31,32,33]. The major PrP^C^ ligands described are molecules related to adhesion and migration processes, such as neural cell adhesion molecule 1 (NCAM1), laminin, and laminin receptors [27]. Indeed, recent data from our group have shown that the modulation of PrP^C^ expression can affect E-cadherin recruitment to the surface and cell migration in glioblastoma stem cells [31], demonstrating a relevant involvement of PrP^C^ in these processes. PrP^C^ also plays an important role in cell adhesion during zebrafish gastrulation [34] and migration of brain endothelial cells [35], forms adherens junction (AJ) with E-cadherin and F-actin in epithelial cells [36] and induces reorganization of the actin cytoskeleton in human T cells [37], among other hallmark features related to the motility of several types of cells.

This review discusses the biological processes involved in cell motility and migration, highlighting the participation of PrP^C^ as a signaling organizer in these mechanisms for the proper functioning of cells under physiological conditions, as well as in the progression of cancer, focusing on PrP^C^ as a player in invasion and metastasis events of several types of neoplasm.

## 2. Prion Protein in Dynamic Cell Movement 

Functional components that actively participate in several aspects of cell motility processes are located on dynamic multi-molecular platforms on the plasma membrane. PrP^C^, a versatile protein with scaffold property, represents a potential molecule able to orchestrate the activity of signaling modules on the cell membrane involved in cellular migration. In this section, we discuss the pivotal role played by PrP^C^ in modulating different motility phenomena, highlighting its interaction with proteins that regulate cell–cell and cell–matrix adhesion, as well as other novel partners in the context of the multi-step migration process.

As previously mentioned, PrP^C^ expression is high in the CNS and PNS, where its function has been extensively studied. The role played by PrP^C^ in the control of synapses, myelination, neuronal survival, and differentiation conceive this protein as a prominent neurotrophic modulator [30,38,39]. Following differentiation, neuron cells attach to the extracellular matrix (ECM) and start to project cytoplasmic extensions of the cell body, called neurites, in order to migrate [40]. PrP^C^ modulates neurite outgrowth and neuronal survival when secreted as a soluble molecule, working as a ligand for signal transduction proteins [30,41]. The neurite growth process, in turn, requires cytoskeleton remodeling, and complete depletion of PrP^C^ leads to β1 integrin aggregation, FA turnover, and increased stability of actin filaments, ultimately resulting in impaired neurite sprouting [42]. FA comprises structures rich in cell adhesion molecules (CAMs) such as integrin, an α/β heterodimeric adhesion glycoprotein receptor that clusters when bound to its ligand, thereby forming multiprotein complexes for intracellular signaling and actin cytoskeleton remodeling [43]. Moreover, FA formation is regulated by Ras homolog family member A (RhoA) activity which, in turn, is modulated by c-Src and focal adhesion kinase (FAK) proteins [44,45]. Additionally, PrP^C^ signaling has been suggested to impact axon guidance.

**Figure 1:**
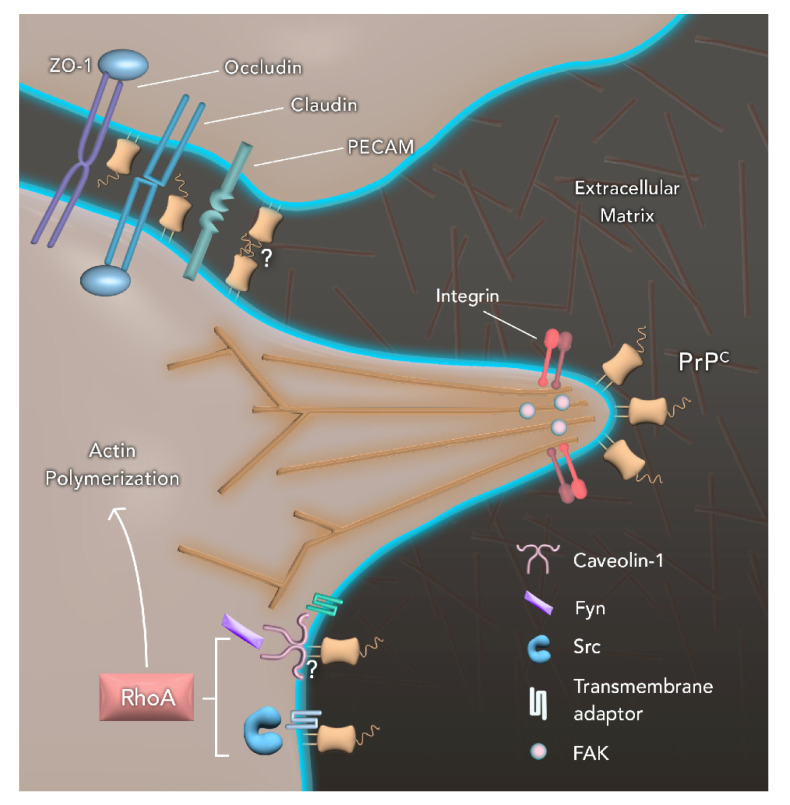
Cellular prion protein (PrP^C^) participates in cell motility through interaction with several partners. PrP^C^ can promote the activation of Fyn in a caveolin-1-dependent manner, although the exact mechanism is unknown. Additionally, PrP^C^ can also modulate the activation of Scr. Since PrP^C^ is a glycophosphatidylinositol (GPI)-anchored protein and all the aforementioned proteins are cytosolic, it is postulated the existence of a transmembrane adaptor that acts as an intermediator. Both Fyn and Src are essential for the regulation of Ras homolog family member A (RhoA) activity. In turn, RhoA has a role in the actin polymerization and assembly of F-actin, as well as the formation of cell protrusions such as focal adhesions (FA) with the presence of focal adhesion kinase (FAK), a central regulator of FA. At the tip of FAs is observed an agglomeration of PrP^C^, as well as integrins and, since PrP^C^ is known to interact with molecules from the extracellular matrix (ECM), it is postulated that both proteins have an important role in adhesion to the extracellular matrix during cell movement. Additionally, PrP^C^ is also known to modulate adhesiveness, being found at the tip of FA and co-localizing with key proteins in cell–cell adhesion domains such as occludin, claudin, PECAM and ZO-1. It is not yet clear if PrP^C^ can interact homophilically with other PrP^C^ molecules from the neighboring cells, although a co-localization is observed.

Growth cones are essential for guiding the process of neurite sprouting, which is fundamental for the morphogenesis of the nervous system [46]. The growth cone per se is divided into two regions: the central domain, rich in microtubules and other associated proteins, and the peripheral domain, rich in actin filaments [47]. The polymerization of those filaments results in *lamellipodia* and *filopodia*-like protrusions with membrane receptors at their tips, sensing a variety of extracellular factors of the environment that act as guidance cues for this process [48]. PrP^C^ was found to be accumulated at the tip of *filopodia* in growth cones and in cell–cell connection sites. It has been observed that the upregulation of the protein in these specific sites is accompanied by growth cone extension and formation of AJ and nanotubes [49]. In agreement, challenging the notion that PrP^C^ knockout (KO) had no major deleterious effects in early mouse embryos [50], Bradikov and collaborators showed that hippocampal neurons lacking PrP^C^ had smaller neurites and growth cones [51]. More specifically, PrP^C^ is able to cluster in reggie membrane microdomains [52], activating the Src-kinase Fyn and Mitogen-activated protein kinase (MAPK), dynamically regulating the polarized transport of membranes and membrane proteins, such as N-cadherin, to the growing tip of neurons [51] (Figure 1). This turnover of membranes and membrane proteins in the growth cone is a fundamental step to the correct targeting and subsequent elongation of neurites towards the right direction of impulse, ensuring correct nerve cell connections [53]. In addition, it is known that the activation of integrin receptors may cause an influx of Ca^2+^ that affects membrane trafficking and reorganization of the growth cone [54]. PrP^C^-KO leads to a reduction in the number and trafficking of vesicles containing N-cadherin, an important component of AJ, diminishing N-cadherin association with reggie microdomains and resulting in impaired downstream signaling, ultimately leading to smaller growth cone and elongation failure [51]. 

PrP^C^ function in the growth cone appears to be dependent, in some instances, on the well-established ability of the protein in binding copper ions. Using focal neurite stimulation, a technology that enables access to the effect of soluble recombinant PrP^C^ molecules released locally in a single growth cone, researchers have described that PrP^C^ can function as a ligand, binding the GPI-anchored form of the protein and initiating a signaling cascade that activates actin remodeling in the periphery of growth cone [55]. More recently, the same research group showed that mutations in distinct copper-binding regions of PrP^C^ impaired neurite outgrowth and growth cone rapid navigation, possibly impacting axonal guidance [56].

The presence of some contrasting evidence on PrP^C^ function for neurite outgrowth is worth noting. A report by Davanathan et al. shows that PrP^C^ may have a negative effect on neurite sprouting through its interaction with Reelin, a glycoprotein with proteolytic activity, and the contactin-associated protein (Caspr), a novel binding partner of PrP^C^ [57]. In this model, PrP^C^ inhibits Reelin’s proteolytic function, protecting Caspr through direct interaction, maintaining its inhibitory effect on neurite growth [57]. These authors argue that this evidence does not contradict previous work that suggests that PrP^C^ influences neurite growth, but rather advocate that PrP^C^ is a regulator of environmental cues of the CNS that balances between neurite outgrowth promotion and inhibition, acting as a positive modulator of this process only in the absence of Caspr activity [57]. A more in-depth study on the signaling behind the observed phenotypes is required to improve the understanding of the proposed role of PrP^C^ in inhibiting neurite sprouting since several different reports have pointed to its role in promoting neurite formation, with strong experimental signaling evidence.

**Figure 2:**
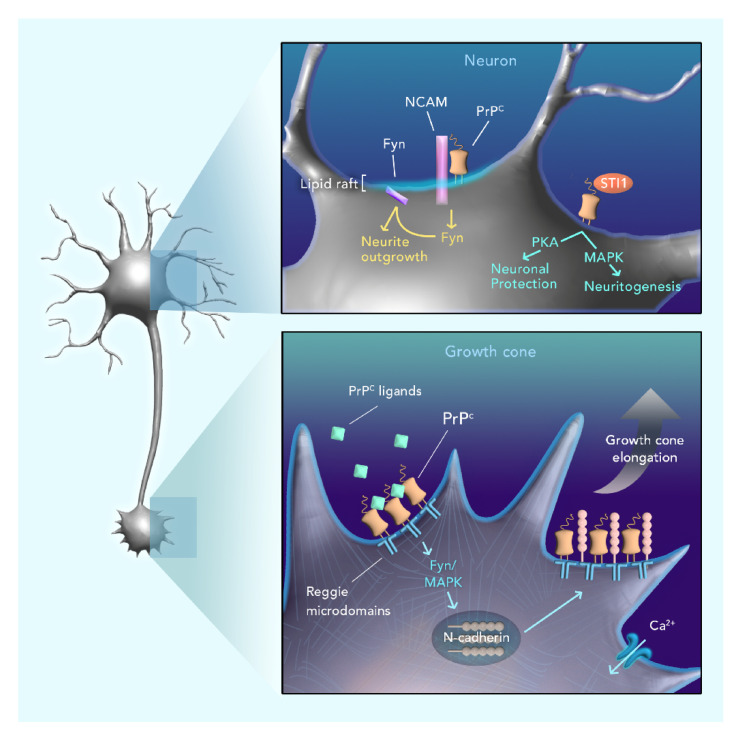
Role of PrP^C^ in neuronal plasticity. Upper panel: PrP^C^ seems to interact with neural cell adhesion molecule (NCAM), promoting the recruitment of Fyn to lipid rafts and stimulating neurite outgrowth. Additionally, PrP^C^ interaction with its well-known ligand, STI1, is able to modulate both Protein kinase A (PKA) and Mitogen-activated protein kinase (MAPK) pathways. The first one is shown to promote neuronal protection, while the second one promotes neuritogenesis. Lower panel: PrP^C^ activated through a ligand or through antibody-mediated crosslinking is able to cluster in reggie microdomains, promoting the activation of Fyn and MAPK. Both of those proteins have an important role in the polarized transport of N-cadherin to regions of growth cone elongation, where it associates with reggie. Additionally, Ca^2+^ intake is also important to the modulation of this process.

As previously mentioned, interaction with a large number of partner proteins is a relevant aspect of PrP^C^ function, not only in the CNS, but also in other tissues. STI1 is among the best-characterized PrP^C^ ligands. This is a well-described co-chaperone that can be secreted by different cells, including astrocytes, and that binds PrP^C^ extracellularly [58]. PrP^C^–STI1 interaction protects cells from the toxic effects of staurosporine-induced cell death through Protein kinase A (PKA) signaling and promotes neuritogenesis through the MAPK signaling pathway [59] (Figure 2). It has recently been demonstrated that specific point mutations that disrupt the PrP^C^–STI1 complex partially or completely impaired neuritogenesis and neuroprotection activity [60], and that STI1 treatment prevented neuronal apoptosis and cell death in a PrP^C^-dependent manner [61]. Although STI1 is a known PrP^C^ partner, the PrP^C^–STI1 complex has not been closely studied in the context of cell motility, despite evidence of its importance. Murine STI1 has been shown to strongly and specifically bind F-actin through its TPR2A and TPR2B domains, which interestingly are also PrP^C^ binding sites [62]. Given the high degree of evolutionary conservation of PrP^C^ and STI1, their wide distribution in different cell types and the data gathered here, the PrP^C^–STI1 complex may regulate processes associated with cell migration, thus requiring further investigation.

Recently, Song and collaborators addressed the role of another possible partner protein, Thymosin Beta 4 (Tβ4), in the deregulation of the cytoskeleton dynamics due to PrP^C^ dysfunction [63]. Tβ4 blocks the polymerization of actin, providing a pool of monomers and functioning on the assembly of the actin filaments [63]. It was observed that Tβ4 mitigates the effects of infection by a PrP amyloidogenic region. Tβ4 treatment increased cell viability, reduced the F-actin/G-actin ratio, and induced the expression of zonula occludens 1 (ZO-1) and occludin, which are proteins found in tight junctions. It is not clear, however, whether Tβ4 acts with PrP^C^ directly, or indirectly through partner proteins [63].

In mammalian brain endothelial cells (BEC), localization of PrP^C^ in cell–cell contacts is dependent on the expression of the protein by two adjacent cells, where it strongly co-localizes with PECAM-1 [35]. PrP^C^ expression increased cell spreading and *filopodia* formation in *Drosophila* and N2a cells [64]. Further, PrP^C^ accumulates at the tips of these protrusions, co-localizing with known FA markers, such as phosphorylated-Src and reggie-1. PrP^C^ knockdown (KD) regulates FA dynamics, leading to a reduction in FA number and an increase in FA size [64]. In polarized intestinal epithelial cells, PrP^C^ was preferentially expressed in cell–cell adhesion sites, interacting with actin and Src proteins [65] (Figure 1). Remarkably, PrP^C^ is not only important for the formation of protrusions and cell–cell and cell–matrix contact in nerve cells, but also for other types of cells and tissues.

In zebrafish, PrP^C^ was expressed specifically in sites of trans-homophilic interactions (PrP^C^–PrP^C^) [34]. Zebrafish contains the PrP-1 and PrP-2 genes, which are duplicated PrP genes as compared with mammalian PrP^C^ [66]. PrP-1 is ubiquitously expressed at early midblastula stages, while PrP-2 transcripts reach high expression levels in the brain around 30 h post-fertilization [34]. PrP-1 KD in zebrafish embryos resulted in developmental arrest, and this phenotype is explained by the loss of embryonic cell adhesion during gastrulation [34]. Additionally, PrP^C^ KD interfered in the cellular localization of the E-cadherin and β-catenin migration-related proteins and in the disorganization of F-actin in a Ca^+2^-dependent pathway in different model organisms [34], once more indicating evolutionarily conserved cell adhesion and motility roles. Accordingly, Huc-Brandt and collaborators reported that, during the collective migration process in zebrafish primordium and in neuromast formation, PrP-2 KO results in E-cadherin and β-catenin mislocalization and loss of cell cohesion [67].

In 2011, a study reported the first direct evidence of PrP^C^ modulation in the migration of mouse brain microvascular endothelial cells (BMVEC). A study showed that siRNA downregulation of PrP^C^ resulted in a reduction of approximately 18% in migrating cells compared with controls, covering an area approximately 9% smaller [68].

Both in zebrafish and mammals, one way through which PrP^C^ modulates cell adhesion and controls dynamics of the actin cytoskeleton is by stimulation of Src-related kinases and its targets. In epithelial cells of the digestive tract, PrP^C^ forms AJ with E-cadherin and F-actin while also co-localizing with Src-kinase [36]. In human enterocytes, PrP^C^ interacts with proteins of the Src kinase family, such as Fyn and Src. PrP^C^ activates Fyn through a caveolin-1-dependent coupling [69] and Src through direct physical interaction [36] (Figure 1). Moreover, in human T cells, PrP^C^ can induce reorganization of the actin cytoskeleton through the Src-related kinases Fyn and Lck and increase the levels of intracellular Ca^2+^ [37]. In zebrafish embryos, PrP-1 buildup at cell contacts elicited similar signaling events [34]. However, in lymphocytes, activated PrP^C^ was found in reggie raft microdomains instead of caveolae [37]. Therefore, the spatial localization of PrP^C^ in microdomains of the plasma membrane might dictate the distribution of Src family signaling partners and consequent activation of different cellular events.

Another way PrP^C^ appears involved with cell–cell adhesion is through interactions with desmosomal proteins, and this has been demonstrated both in enterocytes and BMVEC [70,71]. Intestine from PrP^C^ KO mice and Caco-2/TC7 enterocytes showed increased paracellular permeability and impaired cellular junctions [71]. Cultures from PrP^C^ KD cells exhibited altered morphology and cell junctions, in addition to decreased levels of E-cadherin and the desmosomal proteins desmoplakin, plakoglobin, claudin-4, occludin, ZO-1 and tricellulin at cell contacts [71]. Interestingly, PrP^C^ levels were decreased at cell junctions in epithelia from the colon of patients with Crohn’s disease or ulcerative colitis [71]. In BMVEC, PrP^C^ KD decreased the expression of occludin and claudin-5, leading to augmented permeability in monolayer cultures [70].

PrP^C^ also seems to affect protein interactions in the plasma membrane through the regulation of integrin microclustering and adhesion during monocyte migration. In order to migrate through the endothelium, a process also known as transendothelial migration (TEM) is used, where integrins present in microclusters in the leukocyte plasma membrane bind endothelial ICAM-1 and VCAM-1 receptors, generating strong adhesion to the endothelium [72]. This strong adhesion allows leukocytes to withstand flow-induced detachment and initiate tissue extravasation [72]. This process requires integrins to cluster upon ligand binding, which intensifies avidity and reinforces adhesiveness. Integrin clustering, in its turn, can only occur after its release from the actin cytoskeleton [72,73].

PrP^C^ seems to regulate ligand-induced changes during integrin activation, thus controlling β1-integrin adhesion. PrP^C^ silencing induces cofilin 1 inactivation. This inactivation occurs through phosphorylation on cofilin 1 Ser3 by LIM kinase, which is activated by RhoA [74]. RhoA is able to bind to actin-binding proteins and regulate the assembly of F-actin and the formation of a *lamellipodium*, a cytoplasmic protrusion involved in cell locomotion [75]. Cofilin 1 severs actin filaments, in addition to depolymerizing F-actin, generating new actin monomers for polymerization [76]. Therefore, in PrP^C^-deficient cells, the release of activated integrins from the cytoskeleton is prevented, because RhoA-induced cofilin inactivation sustains the stabilization of actin filaments. Ultimately, this hinders integrin microclustering and avidity for its endothelial targets [74]. This is supported by findings of RhoA activation and consequent cofilin inactivation in PrP^C^-deficient neuronal cells, which increase cytoskeletal stability [77]. In line with this, primary rat hippocampal neurons overexpressing PrP^C^ showed increased cofilin activation and formation of actin-cofilin rods [78], whereas mice overexpressing PrP^C^ showed altered ex vivo thymocyte laminin-induced migration [79].

Moreover, PrP^C^ silencing also altered uropod formation in monocytes possibly by inhibiting phosphorylation of proteins of the ezrin, radixin and moesin (ERM) family. The uropod is a structure with adhesive properties that forms on one edge of the migrating leukocyte during cell polarization. This specialized structure contains cholesterol-rich membrane domains, β1 integrins, and other adhesion receptors, in addition to ERM proteins [80]. In order to migrate, the leukocyte detaches and retracts this uropod, and this process occurs under RhoA control [80]. PrP^C^ silencing alters RhoA activation and reduces ERM phosphorylation with consequent inhibition of uropod formation, since ERM protein activation is essential for this process [74]. As a result, PrP^C^-deficient monocytes show increased chemotaxis and motility on β1 integrin ligands [74].

In the CNS, there is evidence of possible interactions of PrP^C^ with NCAM1 related to physiological cell motility and migration. PrP^C^ and NCAM seem to bind to each other on the plasma membrane, and NCAM could be one of the transmembrane factors linking PrP^C^ and Fyn on N2a cells, since they lack expression of caveolin-1 [81]. NCAM and the L1 cell adhesion molecule (L1-CAM) also take part in neurite outgrowth. More specifically, some members of the Ig superfamily of CAMs can activate important pathways through FGFR receptors and PTKs of the Scr family, leading to tyrosine phosphorylation and thereby assisting in growth cone guidance [82]. Moreover, it has been demonstrated that PrP^C^ and NCAM1 interaction leads to the recruitment of Fyn to lipid rafts and stimulates outgrowth of neurites (Figure 2). This interaction has also been observed in the mouse brain [83], N2a cells [84], and rat cerebellar granule cells [85].

Another role played by this PrP^C^–NCAM1 interaction was revealed for epithelial–mesenchymal transition (EMT). In EMT, epithelial cells develop features of mesenchymal cells towards a migratory phenotype, thus losing cell–cell adhesion structures and polarity [86]. It has been demonstrated that NCAM1 undergoes polysialylation during EMT, and this is hindered by PrP^C^ deficiency [87]. Moreover, this perturbation was caused by decreased transcription of the polysialyltransferase gene ST8SIA2, possibly through transcriptional regulation by β-catenin [87].

Furthermore, the redistribution of NCAM1 and recruitment to caveolae or raft-like domains is an early step of EMT during gastrulation of human embryonic stem cells. This occurs concomitantly with stimulation of p59Fyn (from the Src family kinase), activation of FAK, and the association of integrin-mediated FA [88]. In PrP^C^-1-deficient zebrafish embryos, blastodermal margin cells showed slower migration, while PrP^C^-1-deficient blastomeres showed impaired ability to re-establish cellular contacts. PrP^C^-1 in normal zebrafish embryos, however, increased p59Fyn at cell contacts, in addition to modulating E-cadherin through regulation of its dynamics from intracellular stores to the plasma membrane. It is noteworthy that this altered phenotype of PrP^C^-1-deficient zebrafish embryos was rescued with the introduction of mammalian PrP^C^ [34].

Moreover, expression of PrP^C^ and NCAM1 increases substantially during early EMT induction in mice. In the same model, PrP^C^ increased NCAM1 polysialysation during EMT via modulation of ST8SIA2 transcription [87].

Thus, the physiological roles of PrP^C^ in cell migration seem to occur through direct binding to its targets or via indirect activation of signaling kinases. Hitherto, the main players described in PrP^C^-mediated cell adhesion are those related to Src kinases and adhesion proteins in the plasma membrane, and β-catenin in the cytosol and nucleus, regulating gene expression. Finding novel PrP^C^-collaborating molecules or even attributing new roles to known important partners in the modulation of cellular motility, such as STI1, can bring important information for understanding this process, not only in the physiological context, but especially the contribution of PrP^C^ in pathological processes such as tumor cell migration, which are addressed in the following section.

## 3. Cellular Prion Protein in Cancer Cell Motility

Cell motility mechanisms are quite relevant for cancer survival and are orchestrated by a variety of molecules, including prion protein. The processes of invasion and metastasis allow cancer cells to spread throughout tissues [89], which hinders surgical resections and therapy [90,91,92]. These malignant behaviors are associated with poor prognoses and higher recurrence rates of several cancer types, such as colorectal adenocarcinoma [93], glioblastoma [94], metastatic gastric cancer [95], and melanoma [96]. Strikingly, metastases have been linked to approximately 90% of cancer-related deaths [89]. Tumor resection is a common approach in cancer treatment [97,98,99,100], although data from the literature demonstrate that attempting to resect a tumor may contribute to recurrence and to the cancer capability of forming metastases [101,102]. Concerning these issues, many studies have been focusing on understanding the motility mechanisms of cancer cells and molecules that may be involved in these processes.

Cancer cells can migrate and invade adjacent tissues, and the expression and secretion of proteins related to cell–cell adhesion and ECM degradation are crucial to the invasion and metastasis development during malignant cancers [103]. Invasion occurs when cells penetrate the surrounding tissue, or stroma, through the extracellular matrix and cell–cell interactions [103]. Once these cells cross the basement membranes, they can progress to intravasation of vessels, into the vascular or lymphatic circulation, characterizing the beginning of metastasis. In the next step, the extravasation into the lumen of the vessels of the circulatory system lead cancer cells to establish new tumors (micrometastasis) in any other tissue or organ in the body, generating new secondary and tertiary tumors (colonization) [104].

Mechanisms of invasion and metastasis are closely related and use similar strategies of protein interactions and secretion of proteases, both being considered as a unique hallmark of cancer [105]. These proteins include CAMs such as those from the cadherin and integrin families, which are responsible for ECM interactions [105]. For example, E-cadherin can modulate cell–cell interaction through β-catenin modulation and, in many cancers, the loss of E-cadherin expression promotes invasion and metastasis, which can be associated with EMT processes [104,106,107,108]. On the other hand, N-cadherin expression is increased in several tumors [104,109]. NCAM is another example of a molecule whose expression is related to invasion and metastasis of some types of neoplasms [105,110]. Furthermore, there are many subtypes of integrins expressed in several cancer cells, especially in carcinomas. They can shift the types of integrins expressed on the cell surface according to the ECM, thus facilitating migration and, consequently, invasion and metastasis [105,111]. Additionally, with adhesion molecules, expression of extracellular proteases is crucial to degrade components in ECM to facilitate motility, which can be expressed not only by cancer cells, but also by stroma and inflammatory cells present in the surrounding tissue [105,112]. Indeed, this crosstalk between tumor cells and stroma is extremely important for tumor invasion–metastasis cascades. For example, mesenchymal stem cells can secrete factors that increase invasion and macrophages can secrete matrix-degrading proteases as metalloproteinases, facilitating the intravasation to circulatory vessels [104,113,114,115].

Briefly, the tumor microenvironment is rich in cell–cell and cell–ECM interactions, leading to a close relationship between components in these niches. As such, they work together to facilitate cell migration, promoting invasion and metastatic processes and, consequently, cancer progression.

PrP^C^ has been implicated in the cell motility of several cancer types, including colon and colorectal cancer. Some cells within colorectal carcinoma express PrP^C^ and are spread across the parenchyma of the tumor, albeit they are concentrated mainly in the invasive front of cancer [116], which, in this case, is the region where the majority of EMT occurs [116]. Consistently, it has been shown that PrP^C^ induces EMT in colon adenocarcinoma and colorectal carcinoma cell lines, modulating protein expression levels of E-cadherin and N-cadherin as well as the translocation of β-catenin [116]. Moreover, PrP^C^ is also involved in the metastatic potential of these cells, because it triggers the Fyn-SP1-SATB1 pathway to promote metastasis [116]. Remarkably, the normal tissue that surrounds colorectal carcinoma shows low PrP^C^ expression compared with the cancerous tissue [116]. Additionally, in colorectal adenocarcinoma cell lines, PrP^C^ interaction with STI1 was related to cell migration and invasion by activating ERK1/2 signaling [93]. Data from the literature have demonstrated that some colorectal cancer lineages exhibit a subpopulation of cancer stem cells that express PrP^C^ and are positive for the stem cell marker CD44 [117]. In these cancer stem cells, the transcription factor Twist acts downstream of PrP^C^ and mediates EMT [117]. Furthermore, it has been demonstrated that PrP^C^ interacts with the ECM protein laminin, leading to activation of the MAPK cascade and induction of EMT [117]. In PrP^C^-overexpressing colon cancer cell lines, it was observed that this protein is involved in cell adhesion to fibronectin and collagen in the ECM [118]. Moreover, PrP^C^ augments both the growth and the motility of these cancer cells [118].

Besides colon and colorectal cancer, PrP^C^ also plays important roles in the motility of pancreatic cancer cells. Normal pancreatic tissues do not express PrP^C^, whereas pancreatic ductal adenocarcinoma (PDAC) cell lines express an alternative form of PrP^C^—the so-called pro-PrP^C^, which retains a GPI-anchor peptide signal sequence that crosses the plasma membrane [119,120,121]. In these PDAC cell lineages, this modification enables PrP^C^ to interact with certain molecules such as filamin A (FLNa) [119,120,121] and Notch1 [119], forming multiprotein complexes on the cellular membranes [119]. The interaction between PrP^C^ and Notch1 upregulates the latter, stimulating cell growth and invasion [119]. Importantly, PrP^C^ expression is directly associated with the invasive capacity of this pancreatic cancer [121]. Yet, the interactivity between PrP^C^ and FLNa has implications on the motility of PDAC cell lines [120].

In melanoma, PrP^C^ also exists in the pro-PrP^C^ form, and in FLNa deficient melanoma cell lines, modulations in the *PRNP* gene expression affect cell migration [122]. In addition, PrP^C^ modulates actin polymerization through Akt and HSP27 [122].

PrP^C^ has also been associated with the invasive and metastatic processes of gastric cancer. PrP^C^ is highly expressed in metastatic gastric cancer and it has been observed that this protein contributes to the adhesive capability of gastric cancer cell lines [123]. In addition, it has also been shown that PrP^C^ plays a role in gastric cancer invasion through the ERK1/2 pathway [29,123], as well as in MMP11 expression at both mRNA and protein levels [123]. Concerning the ability of the gastric tumor to metastasize, PrP^C^ is related to gastric cancer metastasis to the liver [123] and lymph nodes [124].

Regarding lung cancer, the *PRNP* gene is upregulated by NFIL3 in invasive lung adenocarcinoma (ILA) cell lines [90] and, at the protein level, PrP^C^ expression is widely observed in invasive tumors, but not in in situ tumors [90]. Furthermore, *lamellipodia* regions of ILA cell lines show augmented PrP^C^ expression, and this protein modulates the levels of active Rac1 and the migration of these cells [90]. PrP^C^ has been implicated in the metastatic capability of ILA in in vivo assays, and PrP^C^ interaction with the JNK pathway induces the motility of ILA cell lines [90]. In addition, NFIL3 and PrP^C^ act towards cancer cell migration [90].

In breast cancer cell lines, PrP^C^-overexpressing cells demonstrated an intensified ability to migrate and invade, without alterations in cancer cell proliferation [124]. Furthermore, it has been shown that a higher expression of PrP^C^ upregulates MMP9 at mRNA levels and, consequently, protein levels, by triggering the NF-κB and ERK pathways, which may contribute to the motility of this cancer [124]. Moreover, multidrug-resistant breast cancer cell lines show increased PrP^C^ and CD44 expressions and interactivity between them, with the latter modulating PrP^C^ [125]. In addition, the interaction held by these two proteins is involved in cell migration mechanisms through the EGFR, CD147, MMP2 and MMP9 proteins, which act as downstream effectors [125].

Among gliomas, glioblastoma multiforme (GBM) is a highly invasive and aggressive tumor [94] that presents PrP^C^ upregulation [33]. It has been demonstrated that PrP^C^ may be involved in the recruitment of E-cadherin to the plasma membrane of GBM stem cells (GSCs) and, in addition, PrP^C^ was shown to influence the localization of β-catenin [31]. Additionally, PrP^C^ was associated with the adhesion and mobility of GSCs, given that changes in PrP^C^ expression affect the migration of these cells on laminin as well as the expression of integrin α6 [31].

In neuroblastoma cell lines, expression of PrP^C^ is involved in cell aggregation and PrP^C^-mediated cell adhesion does not depend on cations [126].

Interestingly, whereas some non-neoplastic tissues present low PrP^C^ expression, this protein is upregulated in cancer tissue samples [116,119]. Moreover, PrP^C^ also shows higher expression in invasive and metastatic cancers compared with non-invasive and non-metastatic ones [90,123].

In summary, these data support an important role for PrP^C^ in cell motility processes of several cancer types, since PrP^C^ has been widely shown to be related to cancer cell migration, invasion and metastasis, being involved in the recruitment of molecules and signaling platforms, in the modulation of the expression of genes and proteins, and in the activation of intracellular pathways in tumor cells (Table 1). Finally, these data point to PrP^C^ as a potential target for cancer treatments, given that its targeting may reduce the malignant behavior of several tumors.

## 4. Perspectives and Conclusions

Cell motility is an important process to the development, maintenance and healing of all tissues, and is essential for the normal functioning of the body. These processes require the transient formation of multiprotein complexes to modulate different signaling cascades which, together, lead to a close-fitting regulation of cellular and molecular phenotypes. As a scaffold protein, PrP^C^ functions as a promiscuous molecule that is able to interact with several partners and, as discussed throughout this review, orchestrates many signaling pathways involved with cell motility. This PrP^C^ scaffolding property might favor protein recruitment and higher-order assemblies into a dynamic molecular platform on the plasma membrane and provide an efficient and reversible mechanism to control intercellular signaling pathways.

The seemingly controversial roles of PrP^C^, such as neurite growth, axon guidance and leukocyte migration, could be explained by the dependence of PrP^C^ on the cell context. For instance, despite the reports supporting PrP^C^ function in neurite sprouting, a mechanism proposed in 2010 shows that, depending on the specific binding partner of PrP^C^ on the cell membrane, it may or may not influence protrusion formation in growth cones [57]. These findings highlight even further the role of PrP^C^ as a sensor of environmental cues, functioning as a key molecule in dynamic control of molecular platforms, in the CNS and other tissues, as well as in health and disease, to trigger different outcomes for the cell.

Although PrP^C^ partners are mostly the same in different tissues—β1-integrin and Src kinases, for example—the balance between upregulated and downregulated signaling pathways will ultimately dictate the function of PrP^C^ in the cell at a given context. In several processes described in this review, however, there are still gaps of knowledge and unknown binding partners of PrP^C^ to explain its effects on cell motility. Thus, the characterization of novel ligands assigned to signaling propagation of PrP^C^ scaffolding platform is of utmost importance to understand PrP^C^ physiological role during cell movement.

In addition, PrP^C^ can also be co-opted by tumoral cells to ensure their survival. Through cell motility processes, tumor cells are capable of invading and metastasizing to other organs and, although the exact percentage is still a debate, a recent study concluded that metastasis could be responsible for more than 65% of cancer deaths [127], while others claim it could be as high as 90% [89]. PrP^C^ expression is upregulated in several types of invasive and metastatic cancers [90,123]. Thus, an intriguing parallel can be established between PrP^C^ physiological and cancer-related functions. While preferential localization of PrP^C^ in specific membrane microdomains favors its interaction with Fyn, supporting proper axon guidance [51], actin cytoskeleton reorganization [37], and N-cadherin transport in the plasma membrane [53], PrP^C^ is also involved in the promotion of metastasis through Fyn activation [116]. Additionally, F-actin and PrP^C^ bind STI1 through the same domain [62], and several lines of evidence point to the promotion of neuroprotection through PrP^C^–STI1 interaction [59,60,61], while the same interaction was associated to cancer cell migration and invasion through ERK1/2 activation [93]. These are some of the examples of physiological PrP^C^-binding preferences that cancer cells might hijack to improve tumor maintenance, invasion and migration.

Furthermore, as discussed in this review, cancer cells appear to take advantage of the PrP^C^ role in cell motility through its binding to N-cadherin and E-cadherin to promote EMT and increase tumor invasion and aggressiveness. In the context of increased survival and proliferation seen in cancer cells, the PrP^C^ function in cell motility seems to be enhanced. Moreover, with the PrP^C^ scaffolding properties facilitating cell signaling, cancer cells are able to invade and spread. Given the functional plasticity of PrP^C^ and its scaffold feature, a deep understanding of its role in the organization and activity of higher-order molecular modules on the cell membrane, in the context of space and time, is imperative to unveil its function as a signaling organizer. Finally, these data demonstrate the importance of uncovering PrP^C^ partners and unveiling their role in motility-related processes, as well as the impact of this molecule as a target for the development of new and effective therapies against metastatic cancer.

## Figures and Tables

**Table 1:** List of tumors along with respective PrP^C^ functions associated with the modulation of invasion and metastasis process.

Types of tumor	PrP^C^ function in tumor cell motility
Colorectal	PrP^C^ interacts with STI-1 to activate ERK1/2 and modulate migration and invasion [93]
	PrP^C^ interacts with laminin to activate MAPK pathway and induce EMT [117]
	PrP^C^ modulates E-cadherin and N-cadherin, as well as translocation of β-catenin to induce EMT [116]
	PrP^C^ has metastatic potential by triggering Fyn-SP1-SATB1 pathway [116]
Colon	PrP^C^ is involved in the cell adhesion to fibronectin and collagen to the ECM [118]
	PrP^C^ modulates E-cadherin and N-cadherin, as well as translocation of β-catenin to the nucleus, inducing EMT [116]
	PrP^C^ has metastatic potential by triggering Fyn-SP1-SATB1 pathway [116]
Pancreatic ductal adenocarcinoma	Pro-PrP^C^ retains a GPI-anchor peptide signal sequence that crosses the plasma membrane [119,120,121]
	Pro-PrP^C^ interacts with Notch1 stimulating cell growth and invasion [119]
	Pro-PrP^C^ interacts with FLNa affecting cell motility [119,120,121]
Melanoma	Pro-PrP^C^ retains a GPI-anchor peptide signal sequence that crosses the plasma membrane [122]
	In FLNa deficient cells, *PRNP* expression affects cell migration [122]
	PrP^C^ modulates actin polymerization trough AKT and HSP27 [122]
Gastric	PrP^C^ contributes to cell adhesion [123]
	Through ERK1/2, PrP^C^ modulates invasion and mRNA and protein levels of MMP11 [123]
Invasive lung adenocarcinoma	PrP^C^ is expressed in invasive tumors, but not in *in situ* tumors [90]
	Enhanced PrP^C^ expression in lamellipodia [90]
	PrP^C^ modulates Rac1 expression and cell migration [90]
	PrP^C^ modulates JNK pathways and induces cell motility [90]
	Together with NFIL3, PrP^C^ acts towards cancer cell migration [90]
Breast	PrP^C^ overexpression intensifies cell migration and invasiveness [124]
	At higher expression PrP^C^ upregulates MMP9 protein and mRNA levels through NF-kB and ERK pathways, contributing to cell motility [124]
	Together with CD44, PrP^C^ is involved in cell migration mechanisms through the proteins EGFR, CD147, MMP2 and MMP9 [125]
Glioblastoma multiforme	PrP^C^ modulates recruitment of E-cadherin to the plasma membrane of GSCs [31]
	PrP^C^ modulates subcellular localization of β-catenin [31]
	Changes in PrP^C^ expression affects cell migration on laminin as well as integrin α6 expression [31]
